# The Cleft Collective: protocol for a longitudinal prospective cohort study

**DOI:** 10.1136/bmjopen-2024-084737

**Published:** 2024-07-05

**Authors:** Amy J V Davies, Kerry Humphries, Sarah J Lewis, Karen Ho, Jonathan R Sandy, Yvonne Wren

**Affiliations:** 1 Bristol Dental School, The Cleft Collective, University of Bristol Faculty of Health Sciences, Bristol, UK; 2 Bristol Medical School, University of Bristol Faculty of Health Sciences, Bristol, UK; 3 Bristol Bioresource Laboratories, University of Bristol Faculty of Health Sciences, Bristol, UK; 4 Bristol Speech and Language Therapy Research Unit, North Bristol NHS Trust, Westbury on Trym, Bristol, UK

**Keywords:** Observational Study, ORAL MEDICINE, PAEDIATRICS

## Abstract

**Introduction:**

Cleft lip and/or palate (CL/P) affects 1 in 700 live births globally. Children born with CL/P and their families face various challenges throughout the child’s development. Extant research is often limited by small numbers and single-centre data. The Cleft Collective, a national cohort study in the UK, aims to build a resource, available to collaborators across the globe, to understand causes, best treatments and long-term outcomes for those born with CL/P, ultimately seeking to enhance their quality of life through improved understanding and care.

**Methods and analysis:**

A longitudinal prospective cohort study of children born with CL/P and their families. Recruitment occurs across the UK and started in November 2013. Recruitment will continue until September 2027 with an estimated final sample of 4822 children born with CL/P (1157 cleft lip including/excluding the alveolus; 2112 cleft palate only; 1042 unilateral cleft lip and palate and 511 bilateral cleft lip and palate). Biological samples are collected from all recruited members of the family. Parental and child questionnaires are collected at key time points throughout the child’s development. Surgical data are collected at the time of surgical repair of the child’s cleft. Consent is obtained to link to external data sources. Nested substudies can be hosted within the cohort. Regular engagement with participants takes place through birthday cards for the children, social media posts and newsletters. Patient and Public Involvement is conducted through the Cleft Lip And Palate Association and Cleft Collective Patient Consultation Group who provide insightful and essential guidance to the Cleft Collective throughout planning and conducting research.

**Ethics and dissemination:**

The Cleft Collective was ethically approved by the National Research Ethics Service committee South West—Central Bristol (REC13/SW/0064). Parental informed consent is required for participation. Findings from the Cleft Collective are disseminated through peer-reviewed publications, conference presentations, newsletters and social media.

**STRENGTHS AND LIMITATIONS OF THIS STUDY**

- The Cleft Collective cohort study will establish one of the largest, prospectively collected data resources and gene banks of children born with cleft lip and palate in the world.
- Comprehensive longitudinal data collection from parents at key time points of the child’s development.
- Multidisciplinary data collection reflecting the multidisciplinary care received by children born with a cleft lip and/or palate in the UK.
- Multicentre recruitment across the whole of the UK.
- As with all cohort studies, there is a risk of loss to follow-up bias and healthy participation bias, with the possibility of syndromic cases not being fully represented.

## Introduction

Cleft lip and/or palate (CL/P) is one of the most common congenital conditions in humans with an estimated prevalence of 1 in 700 live births, which affects around 1000 live births per year in the UK.^1^ Somewhere in the world, every 3 min a child is born with a form of CL/P.^2–4^ Phenotypic expressions comprise cleft lip only; cleft lip including the alveolus; cleft palate only; unilateral cleft lip and palate and bilateral cleft lip and palate. There is evidence to suggest that CL/P subtypes have different aetiologies.^5–7^ Cleft palate only is the most common CL/P subtype, seen in 43.8% of children born in the UK with CL/P in 2022. Cleft lip only and cleft lip including the alveolus, comprised 24.0%; unilateral CL/P 21.6% and bilateral CL/P 10.6%.^1^ The presence of CL/P can be isolated or can occur with additional structural and functional anomalies and approximately 30% of children with CL/P will have a related syndrome.^8^

Previous studies have demonstrated that both genetic and environmental factors play a role in influencing the risk of CL/P.^2 8 9^ Research on environmental risk factors to date has comprised mainly small observational studies which are insufficiently robust for establishing causality or in providing substantial evidence for modifiable factors. Children born with CL/P require surgery and healthcare support into adulthood.^4 10^ The burden on the individual and the health services is considerable.^11^ Adverse outcomes include feeding problems, facial scarring and growth disturbances, speech and dental development. These adverse outcomes may have negative consequences on educational, vocational, social, mental and physical outcomes.^12–17^ The evidence to support preventive interventions and effective treatments is limited as studies are often based on data from a single centre.

In 2012, the James Lind Alliance set out the top 12 priorities for research in CL/P, these included questions regarding the genetic and environmental causes of CL/P; optimum protocols for interventions in surgery, speech and language therapy, audiology, dentistry, orthodontics and psychology and determining those factors associated with educational and personal outcomes.^18^ These priorities are reflected in three key questions often asked by parents after their child has been born with CL/P:

- What caused my baby’s cleft?
- What are the best treatments for my baby?
- Will my baby be OK as they grow up?

To help answer the questions posed by parents and the James Lind Alliance, a cohort study was developed at the University of Bristol, UK. This is uniquely possible within the UK given its large population and provision of cleft care through 11 designated cleft services, creating a UK-wide clinical and research network.^19^

The study is designed to follow the lives of a large group of people to try to understand today’s challenges for those born with CL/P. Our aim is to create the infrastructure and resources necessary to advance our understanding of the causes of CL/P, inform treatment and ultimately improve the lives of children, adolescents and adults with the condition. The cohort study is available as an international resource for clinicians and academics.

## Methods and analysis

### Study design and setting

The Cleft Collective (https://www.bristol.ac.uk/cleft-collective/) is a prospective longitudinal cohort study of children born with CL/P and their families. Participants are recruited to the study by one of the 16 cleft teams, from 11 centralised services, across the UK. The Cleft Collective is split into two cohorts, the birth cohort and the 5-year-old cohort. The birth cohort is further split into two arms, the antenatal arm and the postnatal arm. Families can only be recruited to one cohort; they cannot be recruited to both.

The Cleft Collective has been adopted onto the National Institute for Health and Care Research Clinical Research Network (NIHR CRN) Portfolio (study number 14362).

### Study population

Children born with CL/P (study child), biological mothers, biological fathers or mothers’ partner (collectively known as the parents) and siblings may be recruited to the Cleft Collective. To be eligible for the Cleft Collective, the study child must have been born with CL/P and should be under the care of a UK cleft team at the time of recruitment.

### Recruitment and informed consent

Recruitment to the Cleft Collective started in November 2013 and is currently due to end by 30 September 2027.

**Figure 1:**
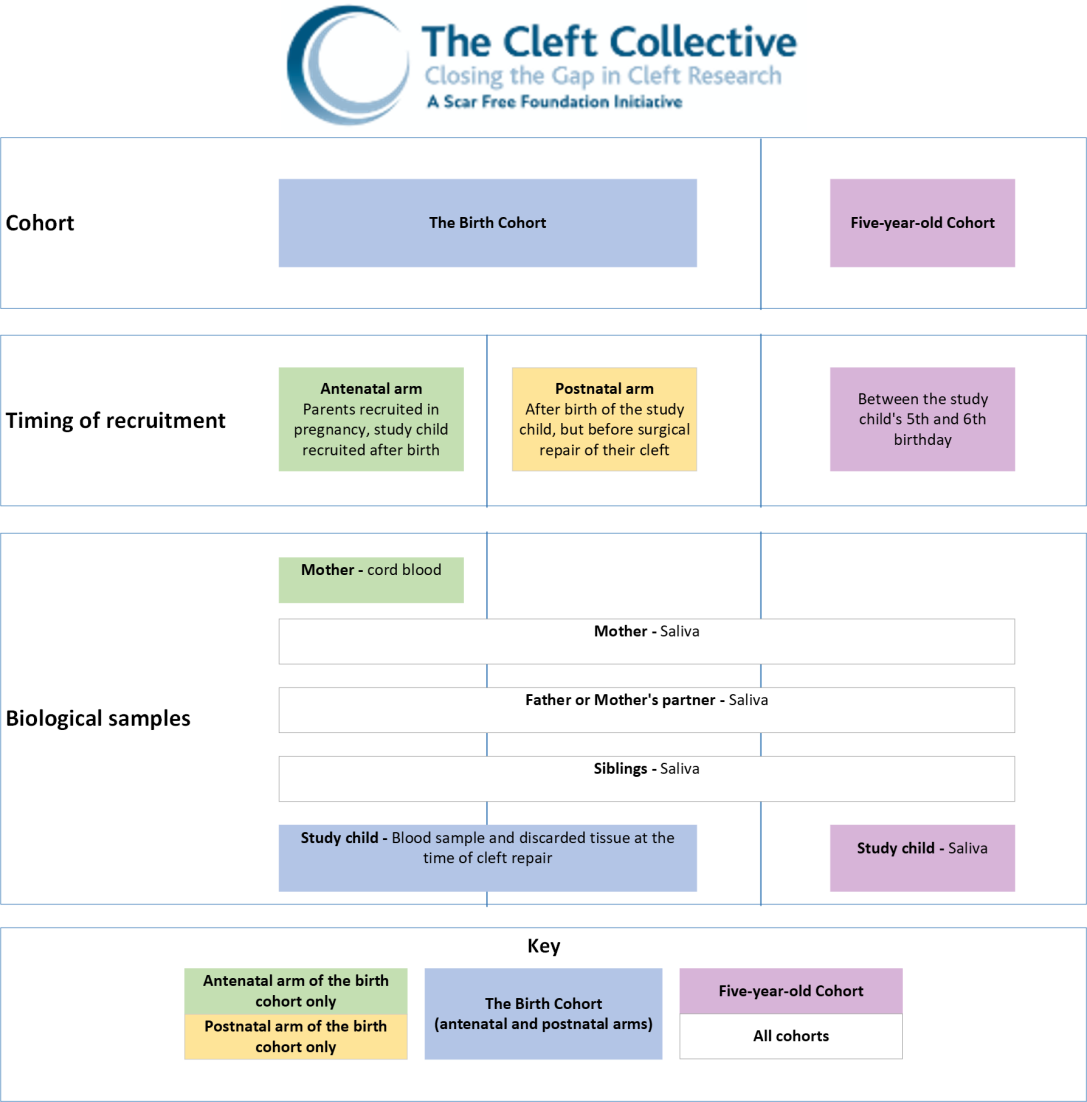
Breakdown of Cleft Collective cohorts, recruitment arms and biological samples.

Families are recruited to the study by a research nurse or a cleft nurse specialist. Parents are provided with an information leaflet about the study and, if they are interested, they are then given a more detailed Patient Information Sheet. If they would like to take part in the study, they must provide informed consent for themselves, the study child and any recruited siblings. Siblings recruited to the study aged 7 years or over are asked to provide assent. Within the antenatal arm of the birth cohort, parents and siblings are recruited during pregnancy and the study child is recruited after birth but before the primary surgical repair of their CL/P. Families recruited to the postnatal arm of the birth cohort are recruited after the birth of the study child but before the primary surgical repair of the study child’s CL/P. Families recruited to the 5-year-old cohort are often recruited at a 5-year-audit clinic, which is part of the study child’s standard care pathway. Families may also be recruited to the 5-year-old cohort study independently of the audit clinic but must be recruited during the year the study child is 5 years old. Figure 1 provides a visual overview of the cohorts and recruitment arms.

### Biological samples

Parents consented to the Cleft Collective donate saliva samples after they have been recruited. Saliva samples are also donated by study children in the 5-year-old cohort and siblings recruited in either cohort. Saliva samples are collected using Oragene 500 DNA Self-Collection Kits (http://www.dnagenotek.com/ROW/products/OG500.html). Residual tissue and blood samples are collected from study children consented to the birth cohort, at the time of the primary surgical repair of their CL/P. Where an expectant mother has consented to the antenatal arm of the birth cohort, a cord blood sample will be collected at the time of delivery. Samples of residual tissue are preserved in RNA*later* Stabilisation Solution. Blood samples are collected with EDTA as the anticoagulant.

All samples are returned to the Bristol Bioresource Laboratories (https://www.bristol.ac.uk/population-health-sciences/research/groups/bblabs/) and are logged on receipt. Oragene kits containing saliva samples are stored at room temperature. Residual tissue in RNA*later* is frozen at minus 80°C. Blood samples in EDTA are processed by centrifugation at 2200g, where g is the relative centrifugal force, for 10 min to obtain white blood cells and plasma aliquots for long-term storage at minus 80°C. DNA is extracted from saliva and blood samples. Samples with sufficient DNA are genotyped.

### Genotyping

Using DNA extracted from the biological samples, genome-wide single-nucleotide polymorphisms genotyping is carried out by the Bristol Bioresource Laboratories using Infinium global screening arrays-24 V.3.0 from Illumina, following manufacturer’s instructions. Physical samples and genotyped data are available within the Cleft Collective resource.

### Parental questionnaires

At the time of recruitment, parents are asked to complete a baseline questionnaire. The baseline questionnaire asks the parent to provide data about their demographics such as ethnicity, education and employment, family life, health and illness at different time points including at conception of the study child, lifestyle, healthcare satisfaction and well-being.

Parents are further asked to complete follow-up questionnaires at key time points of the study child’s development. Within the birth cohort, follow-up questionnaires are sent to parents when the study child is aged 18 months, 3 years and 5 years. Parents within the birth and the 5-year-old cohort are sent follow-up questionnaires when the study child is aged 8, 10, 12 and 15 years. Follow-up questionnaires ask parents to provide data on their child such as information on their health, feeding routines, teeth, development and well-being. Follow-up questionnaires also ask the parent to provide data on their family, lifestyle, healthcare satisfaction and well-being, including validated measures.

### Child questionnaires

Where a parent has consented for their child to receive questionnaires, the study child will be sent a questionnaire at ages 8, 10, 12 and 15 years. Child questionnaires at ages 8, 10 and 12 are shorter than parental questionnaires, with the 8-year-old questionnaire only comprising four pages. Data collected within child questionnaires include data on how the child feels about their cleft, their development and their well-being, including validated measures.

Copies of parental and child questionnaires can be found at https://www.bristol.ac.uk/cleft-collective/professionals/information/questionnaires/

Content and timing of all questionnaires were determined by input from clinical partners, clinical excellence networks, previous studies, the Cleft Lip And Palate Association (CLAPA) and the internal research team. Input and advice sought from clinical partners and clinical excellence networks represent many different disciplines including nursing, psychology, surgery, speech and language therapy, audiology, dentistry and orthodontics. Existing studies provided some of the questionnaire content and these included the Avon Longitudinal Study of Parents and Children^20 21^ and their second generation,^22^ Life study,^23^ Born in Bradford^24^ and the Norwegian Mother and Child Cohort Study.^25^ Many of these are general population studies from which a control sample can be derived. Questions within the questionnaires are often used again at different ages which allows the detection of any trajectories or changes as the child develops.

### Surgical questionnaires

Surgeons at each cleft centre are asked to complete surgical forms at the time of surgical repair or a CL/P-related surgery for children within the birth cohort. Data collected at the time of surgery include the type of surgical procedure being performed, technique used, surgical adjuncts and CL/P phenotype.

Timepoints of questionnaires are provided within table 1.

**Table 1:** Timing of questionnaires

	Parental baseline questionnaires	Parental follow-up questionnaires	Child questionnaires	Language ENvironment Analysis—audio recordings	Parental speech and language questionnaires	Speech and language assessments	Clinical audiology reports	Speech and Language Intervention survey	Surgical forms
Pregnancy	✔								
Post birth	✔								✔^*^
13 months				✔	✔				
16 months							✔		
18 months		✔				✔^†^			
24 months									
3 years		✔			✔	✔	✔		
5 years	✔	✔						✔	
8 years		✔	✔						
10 years		✔	✔						
12 years		✔	✔						
15 years		✔	✔						
Key	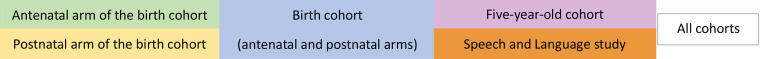								

*Surgical forms are collected at the child’s primary cleft repair and are requested at subsequent cleft related surgeries, which may occur throughout childhood.

†Speech and language assessment forms are collected at the child’s assessment which may be at 18 or 24 months.

A data dictionary detailing all available data is available online and can be found at https://www.bristol.ac.uk/cleft-collective/professionals/access/

### Format of data collection

All questionnaires are available in paper format. Paper questionnaires returned to the Cleft Collective are scanned by an automated document recognition software, Formic Fusion (https://www.formic.com/). Where the software is unable to read parts of the scanned questionnaire, human verification is required. Quality control checks are implemented when cleaning the data. Where responses appear to be invalid, the original questionnaire is checked to confirm the response. If the original response matches the response within FORMIC and it is still considered to be invalid, the response is set to missing. If the original response does not match the response within FORMIC, the response is corrected and an additional 5% of random responses for the offending question are checked. If further mismatches are found, all responses to the offending question are checked, to date we have not needed to implement this full process. Parental follow-up questionnaires, the study child’s 15-year questionnaire and the surgical questionnaire are also available as online versions, using Research Electronic Data Capture (REDCap; https://www.project-redcap.org/), a secure web application for building and managing online surveys.^26 27^ Quality control checks have been added to the online surveys, where a participant enters an invalid response REDCap will inform the participant that their response is invalid and will ask them to re-enter the information. Online questionnaires were introduced to the study in 2020.

### Linkage to external data

At the time of recruitment, parents are asked to provide consent to link the data provided to the Cleft Collective to external data sources. These external data sources include the Department for Education (specifically the National Pupil Database), provided by the Office of National Statistics (ONS), NHS England (specifically Hospital Episode Statistics), the Cleft Registry and Audit NEtwork (CRANE) Database and medical records held by cleft teams and healthcare providers. Consent for linkage to external data is optional, participants who do not provide consent for this aspect of the study may still consent to other parts of the Cleft Collective.

Due to requirements stipulated by the external data owners, linkage will take place on a project-by-project basis. Collaborators wishing to use Cleft Collective data and link it with external sources will need to liaise with the Cleft Collective Project Management Group to seek approval for their project. Collaborators will also be expected to obtain approval with the relevant data linkage providers while working alongside the Cleft Collective study team. Once all approvals have been obtained and relevant contracts have been signed, participants’ data will be linked to the relevant external sources following the data owner’s procedure. Education data will be accessed through the ONS Integrated Data Service. Linked data from other sources will be sent and received via secure encrypted methods or alternative secure methods as determined by the external data owners.

### Cleft Collective Speech and Language study

Nested substudies can be hosted within the Cleft Collective. The largest nested substudy is the Cleft Collective Speech and Language study (CCSL). Children within the birth cohort born with a cleft palate (±cleft lip) can be recruited to the CCSL alongside one parent. Consent is provided for further data collection on the study child’s speech and language development. Data collected within the CCSL include audio recordings using the Language ENvironment Analysis software (LENA),^28^ parental questionnaires about receptive and expressive language at ages 13 and 36 months, speech and language therapist assessment data at ages 18–24 and 36 months, audiology assessment data at ages 16 and 34 months and details on the interventions that the child has received up until the child is aged 5 years and 11 months.

Further information about the CCSL study can be found at https://www.nbt.nhs.uk/bristol-speech-language-therapy-research-unit/bsltru-research/cleft-speech-language-study

### Participant retention

Engagement with participants enables us to provide updates on the study progress and to enhance the retention of participants.^29^ In addition, the Cleft Collective regularly update participants on the studies progress and any research findings using the study website, social media and through newsletters. Study children are sent birthday cards each year on their birthdays to help keep families engaged. As a thank you for the time taken to complete questionnaires, parents and children are sent monetary vouchers.

### Patient and public involvement

In 2012, during the design and set up of the study, the Cleft Collective worked alongside CLAPA, a UK charity supporting people born with a cleft and their families, to run two patient and public involvement (PPI) workshops. The PPI workshops helped inform the design of the Cleft Collective.

PPI involvement continues to inform the Cleft Collective through the CLAPA Cleft Collective Patient Consultation Group (CLAPA CC PCG). The CLAPA CC PCG provides essential guidance to the Cleft Collective throughout planning and conducting research. This informs many aspects of the study including questionnaire and study design. In 2020, the CLAPA CC PCG won the Royal College of Paediatric and Child Health/NIHR Paediatric Involvement and Engagement in Research (PIER ) prize.

### Power calculations

It is estimated that that by the end of September 2027, the Cleft Collective will have recruited 4822 children born with CL/P. It is further estimated that the breakdown of CL/P subtype will include 1157 children born with a cleft lip only and cleft lip including the alveolus; 2112 children born with a cleft palate only; 1042 children born with a unilateral cleft lip and palate and 511 children born with a bilateral cleft lip and palate. At the time of writing this protocol, 4020 children born with CL/P had been recruited (a total of 11 298 participants including study child, mothers, fathers, mother’s partner and siblings). Estimated figures account for attrition as a result of withdrawal from the study. To date, 58 study children and 168 participants overall (inclusive of the study children) have withdrawn from the study (1.4% of study children; 1.5% of all participants). Of these withdrawals, 19 study children and 56 participants overall (inclusive of study children) requested that their data were destroyed (0.5% of all study children; 0.5% of all participants).

As the Cleft Collective is a resource for the academic and clinical community, it is likely that there will be a wide range of current and future research questions proposed by collaborators. Power calculations have therefore been undertaken for a range of possible combinations. Minimum detectable risk ratios have been calculated for within sample analyses (table 2). Where hypotheses are looking to determine whether a cleft-related outcome with a prevalence of 10% is associated with an exposure present in 20% of the cohort, the minimal detectable risk ratio (with 80% power and the probability of a type 1 error at 5%) is 1.28 with a sample size of 4822.

**Table 2:** Minimum detectable risk ratio with an overall sample size of n=4822, 80% power and probability of a type 1 error at 5%

		Prevalence of outcome			
		10%	20%	30%	40%
Proportion of control sample exposed	0.1	1.43	1.32	1.28	1.26
	0.2	1.28	1.21	1.18	1.17
	0.3	1.21	1.16	1.14	1.13
	0.4	1.17	1.12	1.11	1.10
	0.5	1.13	1.10	1.09	1.08

Further power calculations have been undertaken to provide minimum detectable ORs when using a case–control design. An example of such analysis may include exploring the association between environmental risk factors and presence of CL/P. The control sample would need to be obtained from an additional source. For analyses with CL/P subtypes as the outcome, the estimated proportion of each CL/P subtype within the sample was calculated from the breakdown of cleft subtype reported in the CRANE 2022 annual report.^1^ Where hypotheses are looking to determine whether an environmental risk factor, present in 20% of the control population, is associated with bilateral CL/P using a case–control design and a case-to-control ratio of 1:4, the minimum detectable OR (with 80% power and the probability of a type 1 error at 5%) is 1.39 with a case sample size of 511(table 3).

**Table 3:** Minimum detectable OR with a cleft subtype case sample and non-cleft control sample, 80% power and probability of a type 1 error at 5%

		Case-to-control^*^ ratio			
		1:1	1:2	1:3	1:4
		Cleft lip only (n=1157)			
Proportion of control sample^*^ exposed	0.1	1.44	1.39	1.37	1.33
	0.2	1.32	1.29	1.27	1.25
	0.3	1.28	1.26	1.24	1.22
	0.4	1.27	1.24	1.23	1.21
		Cleft palate only (n=2112)			
	0.1	1.31	1.28	1.27	1.24
	0.2	1.23	1.21	1.20	1.18
	0.3	1.20	1.18	1.17	1.16
	0.4	1.19	1.17	1.16	1.15
		Unilateral cleft lip and palate (n=1042)			
	0.1	1.46	1.42	1.39	1.35
	0.2	1.34	1.31	1.29	1.26
	0.3	1.30	1.27	1.25	1.23
	0.4	1.28	1.25	1.24	1.22
		Bilateral cleft lip and palate (n=511)			
	0.1	1.70	1.62	1.58	1.52
	0.2	1.51	1.46	1.43	1.39
	0.3	1.45	1.40	1.38	1.34
	0.4	1.42	1.38	1.36	1.32

*Control sample would need to be obtained from an additional source.

### Statistical analyses

The Cleft Collective is a resource available to clinicians and researchers. When a project proposal is submitted to access the resource, collaborators are asked to outline their project specific planned statistical analyses.

### Data deposition and curation

All data held by the Cleft Collective are stored securely at the University of Bristol and in accordance with the University of Bristol’s information security policies. The Cleft Collective is a resource available to clinicians and researchers worldwide, data will be made available to collaborators subject to an approved project proposal by the Cleft Collective Project Management Group and ethical approval by an affiliated ethics board. All data supplied to collaborators will be pseudonymised with a unique collaborator identifier. Any curation of data by collaborators must be returned to the Cleft Collective resource at the end of the collaborator’s project. Any data transferred will be encrypted to a minimum Advanced Encryption Standard (AES) 256-bit.

Details on how to access the Cleft Collective resource can be found at https://www.bristol.ac.uk/cleft-collective/professionals/access/

## Ethics and dissemination

Ethical approval for the Cleft Collective was provided by the National Research Ethics Service committee South West—Central Bristol (REC13/SW/0064). The Cleft Collective is sponsored by the University of Bristol. Parental informed consent is required for participation.

Findings from the Cleft Collective resource will be disseminated through peer-reviewed publications and through national and international conference presentations, both oral and poster. Collaborators using the Cleft Collective resource for their research may also publish preprints of their papers using preprint servers such as Medrxiv or Biorxiv subject to their target journal guidance. Papers published will be promoted through the Cleft Collective website, newsletters and through social media.
